# Separation and Structural Characterization of a Novel Exopolysaccharide from *Rhizopus nigricans*

**DOI:** 10.3390/molecules27227756

**Published:** 2022-11-10

**Authors:** Zhang Li, Jianhua Li, Xuan Xu, Zhen Luo, Jiayi Sun, Hongyun Wang, Chunyan Liu, Xiuwen Ni, Jianqi Sun, Jun Xu, Kaoshan Chen

**Affiliations:** 1 Jiaxing Blood Center, Jiaxing 314001, China; 2 School of Life Science, Shandong University, Qingdao 266237, China; 3 Anhui Provincial Engineering Research Center for Polysaccharide Drugs, School of Pharmacy, Wannan Medical College, Wuhu 241002, China; 4 Jiaxing Xiuzhou District Maternal and Child Health Hospital, Jiaxing 314031, China

**Keywords:** *Rhizopus nigricans*, exopolysaccharide, structural characterization, antioxidant activity

## Abstract

The present study aims to analyze the structural characterization and antioxidant activity of a novel exopolysaccharide from *Rhizopus nigricans* (EPS2-1). For this purpose, EPS2-1 was purified through DEAE-52, Sephadex G-100, and Sephadex G-75 chromatography. The structural characterization of EPS2-1 was analyzed using high-performance gel permeation chromatography (HPGPC), Fourier transform infrared spectroscopy (FT-IR), methylation analysis, nuclear magnetic resonance (NMR) spectra, transmission electron microscope (TEM), and atomic force microscope (AFM). The results revealed that EPS2-1 is composed of mannose (Man), galactose (Gal), glucose (Glc), arabinose (Ara), and Fucose (Fuc), and possesses a molecular weight of 32.803 kDa. The backbone of EPS2-1 comprised →2)-α-D-Man*p*-(1→ and →3)-β-D-Gal*p*-(1→, linked with the O-6 position of (→2,6)-α-D-Man*p*-(1→) of the main chain is branch α-D-Man*p*-(1→6)-α-D-Man*p*-(1→, linked with the O-6 positions of (→3)-β-D-Gal*p*-(1→) of the main chain are branches →4)-β-D-Glcp-(1→ and →3)-β-D-Galp-(1→, respectively. Finally, we demonstrated that EPS2-1 also shows free radical scavenging activity and iron ion reducing ability. At the same time, EPS2-1 could inhibit the proliferation of MFC cells and increase the cell viability of RAW264.7 cells. Our results suggested that EPS2-1 is a novel polysaccharide, and EPS2-1 has antioxidant activity. In addition, EPS2-1 may possess potential immunomodulatory and antitumor activities. This study promoted the application of EPS2-1 as the functional ingredients in the pharmaceutical and food industries.

## 1. Introduction

A polysaccharide is a type of natural polymer of more than 10 monosaccharides connected through glycosidic linkages; it is a class of biopolymers with diverse structures [1]. Natural polysaccharides have been used in the food, cosmetics, and the pharmaceutical industry for many years due to their excellent biological activity. Several natural polysaccharides have been reported to have anti-diabetic, antiviral, anti-aging, and immunomodulatory activities [2], which exist widely in plants, animals, and microorganisms [3].

Previously, scientists have successfully isolated numerous polysaccharides with excellent biological activity from fungi [4]. Some fungal polysaccharides demonstrate antitumor activities. For example, a polysaccharide from mycelia of *Phellinus linteus* could inhibit the proliferation of S-180 sarcoma cells, increase the expression level of Bax and Cytochorme c, and induce apoptosis in cells [5]. Meanwhile, a polysaccharide from *Sanghuangporns vaninii* could inhibit cell viability and cell colony formation of NCI-H460 cells, which induced apoptosis of lung cancer cells [6]. At the same time, many fungal polysaccharides possess immunostimulatory activity. The polysaccharide from *Ganoderma* promoted T lymphocyte proliferation and increased the content of IL-2 and IFN-γ of cells, and this effect was realized mainly by the MAPK signaling pathway [7]. A polysaccharide from *Polyporus umbellatus* could increase the killing potency of natural killer (NK) and lymphokine-activated killer (LAK) cells, and promote the proliferation of T and B cells [8]. In addition, a polysaccharide from the *Oudemansiella radicata* mushroom showed ABTS, DPPH, and hydroxyl radicals scavenging effects, and ferrous ion chelating activity [9]. A polysaccharide from *Cordyceps kyushuensis* showed scavenging ability on hydroxyl radicals, and the capacity for a protective effect against DNA damage [10]. Consequently, separating novel fungal polysaccharides with biological activities can make better use of fungi, which can promote the development of drugs and functional foods.

*Rhizopus nigricans* is a filamentous conjugating fungus, which always parasitizes tomatoes, bread, and other daily food [11]. *R. nigricans* can produce amounts of substances with biological activity, and this species is widely studied in the food and medical industries [12]. In a previous study, our group purified EPS1-1 from the fermentation liquor of *R. nigricans* using a DEAE-Sepharose column, which was eluted with Tris-HCl buffer without NaCl [13]. However, the NaCl-eluted fraction (EPS-2) has not been further studied. EPS1-1 was composed of Glc, Man, Gal, and Fru, the molecular weight of EPS1-1 was 9.7 kDa, which was reported to inhibit the occurrence and development of colon cancer by relieving immunological inflammation [14]. Previous studies showed that EPS1-1 inhibits the malignant process of hepatocellular carcinoma in vitro and in vivo [15]. At the same time, EPS1-1 could inhibit the proliferation of CT-26 cells, also induce apoptosis in CT-26 cells [16]. However, the structural characterization and biological activity of EPS-2 have not been further studied.

In this study, the polysaccharides were obtained from the fermentation broth of *R. nigricans* by ethanol precipitation, deproteinization, and decoloration. After purification using the DEAE-52 ion exchange column, Sephadex G-100 column, and Sephadex G-75 column, the novel polysaccharide (EPS2-1) was purified from EPS-2, then subjected to molecular weight and monosaccharide composition analysis. The precise structure of EPS2-1 was further analyzed by FT-IR, GC-MS, and NMR, and the molecular morphology was determined by TEM and AFM. Furthermore, DDPH, ABTS, hydroxyl radicals, and ferric reducing antioxidant power (FRAP) assays were used to detect the antioxidant activity of EPS2-1. Additionally, CCK-8 assay was used to detect the effect of EPS2-1 and EPS1-1 on cell viability of mouse forestomach carcinoma (MFC) cells, human lung cancer cells, and RAW264.7 cells. This study provided certain directions for the research and development of exopolysaccharides from *R. nigricans*, moreover, our work helped to understand this fungus.

## 2. Results and Discussion

### 2.1. Isolation and Purification of EPS2-1

**Figure 1:**
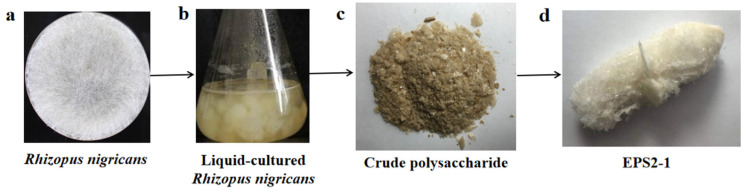
Extraction of crude exopolysaccharides from the fermentation broth of *R. nigricans*.

**Figure 2:**
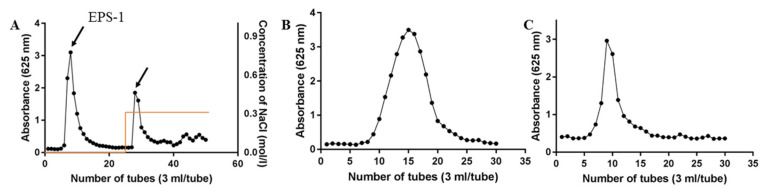
Isolation and purification of exopolysaccharides from the fermentation broth of *R. nigricans*. (**A**) Crude polysccharides were stepwise separated by DEAE-52 ion exchange column; (**B**) Sephadex G-100 column; (**C**) Sephadex G-75 column.

The crude exopolysaccharides from the fermentation broth of *Rhizopus nigricans* were obtained by water extraction, ethanol precipitation, deproteinization, and decoloration (Figure 1) according to previously reported method [13]. The yield of the crude polysaccharides was 196.36 mg/L. The crude polysaccharides were purified by DEAE-52 anion-exchange chromatography column with 0.02 mol/l Tris–HCl and 0.3 M Tris–NaCl solutions, and two fractions (EPS-1 and EPS-2) were obtained (Figure 2A). The structure and biological activity of EPS-1 were studied previously, and EPS-2 was the primary fraction obtained. Further purification using Sephadex G-100 and Sephadex G-75 columns was carried out on EPS-2 (Figure 2B,C). In Sephadex G-75 column chromatography, one peak was observed, and seven tubes (NO. 7–13) were collected and lyophilized, named EPS2-1. The total carbohydrate content of EPS2-1 was 98.56% according to the phenol–sulfuric acid method [17].

### 2.2. Molecular Weight and Monosaccharide Composition

**Figure 3:**
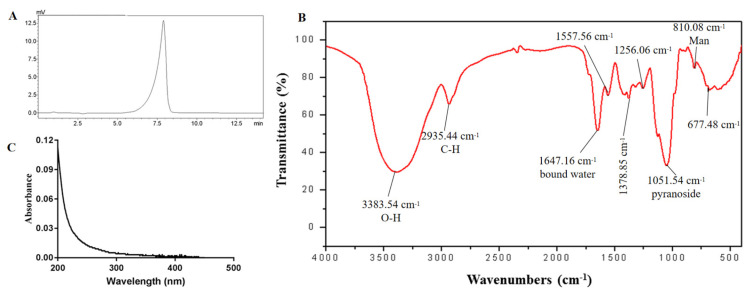
(**A**) HPGPC profile of EPS2-1; (**B**) FT−IR spectrum of EPS2-1; (**C**) Ultraviolet spectroscopy of EPS2-1.

The HPGPC profile of EPS2-1 showed only one narrow symmetrical peak, indicating that EPS2-1 is a homogeneous polysaccharide. The weight-average molecular weight (Mw) of EPS2-1 was estimated to be 32.803 kDa, and the polydispersity ratio was 1.345 (Figure 3A). The monosaccharide composition of EPS2-1 was analyzed using high-performance ion exchange chromatography (HPIC). EPS2-1 was composed of Man, Gal, Glc, Ara, and Fuc with a ratio of 0.519:0.204:0.065:0.031 and 0.029, respectively, which indicated that Man and Gal are the major monosaccharide components (Table 1).

Man and Gal are major monosaccharide components in many fungal polysaccharides, such as the polysaccharide from *Cordyceps militaris* and *Sanghuangporus vaninii* [6]. According to previous studies, EPS1-1 was composed of Glc, Man, Gal, and Fru with a Mw of 9.7 kDa. The molecular weight and monosaccharide composition of EPS2-1 were different from EPS1-1, which indicated that EPS2-1 is a novel polysaccharide. The differences might be due to the different concentrations of NaCl in anion-exchange chromatography.

### 2.3. FT-IR and UV Spectrum

A broad and strong absorption at 3383.54 cm^−1^ was attributed to the O-H stretching vibration, and the absorption at 2935.44 cm^−1^ was due to the C-H stretching vibration. The band at 1051.54 cm^−1^ resulted from the absorption of pyranoside, and the peak at 810.08 cm^−1^ suggested the existence of the Mannose (Figure 3C) [18]. Moreover, the UV spectra suggested that there was no absorption at 260 and 280 nm, indicating the absence of protein and nucleic acid in EPS2-1 (Figure 3B).

### 2.4. Methylation Analysis

Methylation analysis was used to determine the type of linkage of monosaccharide residues. After the methylated product was hydrolyzed, reduced, and acetylated, the composition was analyzed by GC-MS [19]. The methylation analysis results are summarized in Table 2. EPS2-1 was composed of Man*p*-(1→, →2)-Man*p*-(1→, →4)-Glc*p*-(1→, →3)-Man*p*-(1→, →3)-Gal*p*-(1→, →6)-Glc*p*-(1→, →6)-Man*p*-(1→, →2,6)-Man*p*-(1→, →3,6)-Gal*p*-(1→, the molar ratio was consistent with the results of monosaccharide composition. The linkage pattern →2,6)-Man*p*-(1→ and →3,6)-Gal*p*-(1→ indicated that EPS2-1 is a branched polysaccharide (Table 2).

### 2.5. NMR Analysis

**Figure 4:**
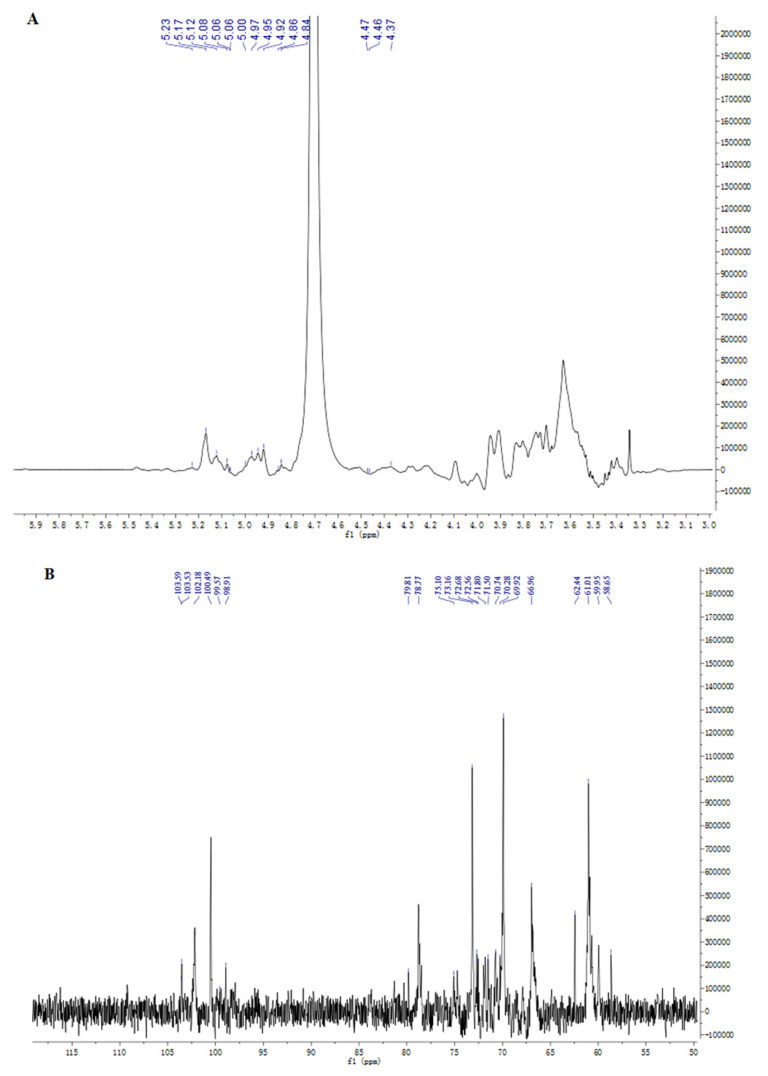
One−dimensional NMR analysis of EPS2-1. (**A**) ^1^H spectrum; (**B**) ^13^C spectrum.

NMR can provide information about the subtle structure of polysaccharides. In this work, 1D NMR and 2D NMR, including ^1^H, ^13^C, HSQC, HMBC, NOESY, and COSY spectra were used to identify the structure of EPS2-1. The chemical shift range of anomeric hydrogen from α-glycosidic bonds is δ 4.80–5.50 ppm in the ^1^H spectra, and the chemical shift range of anomeric hydrogen from β-glycosidic bonds is δ 4.40–4.80 ppm [20]. As shown in Figure 4A, nine chemical shift signals of anomeric protons were found at δ 4.37, 4.46, 4.47, 4.85, 4.91, 4.99, 5.05, 5.15, and 5.23 ppm in the ^1^H NMR spectra, which were named residues A, B, C, D, E, F, G, I, J, and K, repectively [21]. The ^13^C NMR spectra showed that anomeric C-1 signals were detectable at δ 99.1, 99.62, 100.83, 101.1, 101.95, 103.6, 103.84, 104.48, and 104.49 ppm (Figure 4B) [22].

**Figure 5:**
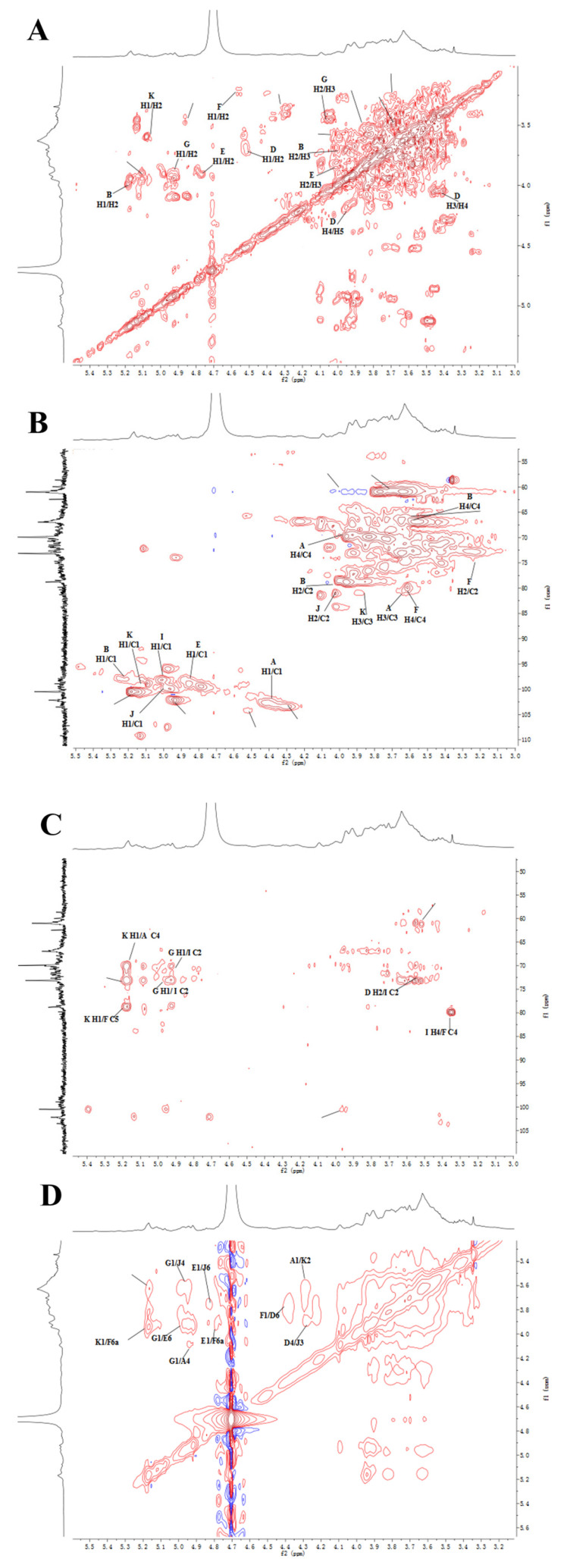

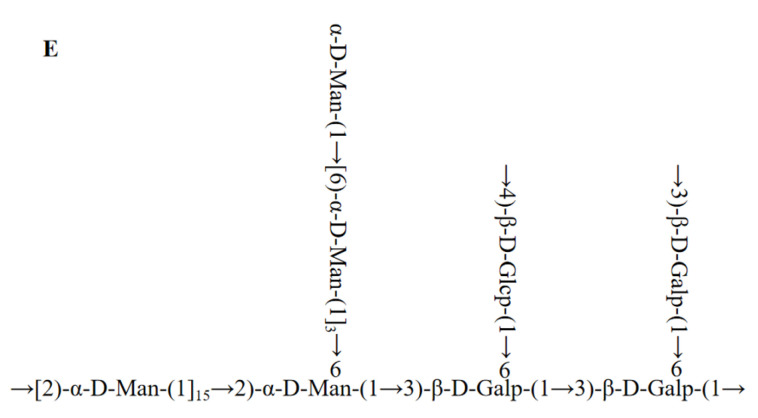
Two-dimensional NMR analysis of EPS2-1. (**A**) COSY spectrum; (**B**) HSQC spectrum; (**C**) HMBC spectrum; (**D**) NOESY spectrum; (**E**) The putative structure of EPS2-1.

Residue B had an anomeric signal at δ 5.23/101.95 ppm in the HSQC spectrum, ^1^H-^1^H COSY spectrum showed a cross peak of H-1 with H-2 (δ 4.01 ppm). The chemical shift of C-2 was assigned to be δ 80.21 ppm in HSQC spectra. Similarly, δ 3.88/71.32, 3.6/68.19, and 3.7/74.5 ppm were classified as H3/C3, H4/C4, H4/C4, and H5/C5, respectively. The carbon signal at δ 80.21 ppm was attributed to the C-2 signal of the (1→2)-linked mannose, therefore, residue B was identified to be →2)-α-D-Man*p*-(1→. The chemical shift of C-6 was shifted to a lower field (δ 67.2 ppm) compared to the unsubstituted C-6, indicating that residue E was →6)-α-D-Manp-(1→. The chemical shift of the heterotopic carbon of residue D was δ 104.49 ppm, which indicated that residue D contained β-D-Gal*p.* At the same time, the downfield chemical shifts of C-3 and C-6 showed that residue D was →3,6)-β-D-Galp-(1→ (Figure 5A,B). Based on the monosaccharide composition, methylation analysis, and literature reports, the primary signal attributions of glycosidic residues in EPS2-1 are shown in Table 3 [23].

HMBC and NOESY spectroscopy were used to analyze various sugar residues’ connection sites and sequence [3]. In the HMBC spectrum (Figure 5C), the anomeric proton of residue B had a cross-peak signal with its C-2, which corresponded to →2)-α-D-Man*p*-(1→2)-α-D-Man*p*-(1→. As shown in the NOESY spectrum (Figure 5D), the anomeric proton of residue B had a cross-peak signal with the H-2 of residue J (BH-1/JH-2), which suggested the existence of →2)-α-D-Man*p*-(1→2,6)-α-D-Man*p*-(1→. The JH-1/DH-3 (δ 5.05/3.68 ppm) indicated the existence of →2,6)-α-D-Man*p*-(1→3,6)-β-D-Gal*p*-(1→. The cross-peak signal at δ 4.99/3.78 ppm was assigned to the correlation between H-1 of residue G and H-6 of residue E, implying glycosidic bond α-D-Man*p*-(1→6)-α-D-Man*p*-(1→ [24]. Another cross-peak signal at δ 4.85/3.77 ppm was assigned to the correlation between H-1 of residue E and H-6 of residue J, which indicated the presence of →6)-α-D-Man*p*-(1→2,6)-α-D-Man*p*-(1→. The cross signal at δ 4.46/3.96 ppm indicated a correlation between H-1 of residue F and H-6 of residue D, implying glycosidic bond →4)-β-D-Glc*p*-(1→3,6)-β-D-Gal*p*-(1→ [25].

Based on the results of methylation and NMR analysis, the putative backbone of EPS2-1 was inferred to be →2)-α-D-Man*p*-(1→2,6)-α-D-Man*p*-(1→3,6)-β-D-Gal*p*-(1→3,6)-β-D-Gal*p*-(1→, and the side chains of EPS2-1 were α-D-Man*p*-(1→6)-α-D-Man*p*-(1→ linked at O6-position of →2)-α-D-Manp-(1→, →4)-β-D-Glc*p*-(1→ linked at O6-position of →3)-β-D-Galp-(1→, and →3)-β-D-Galp-(1→ linked at O6-position of →3)-β-D-Galp-(1→ (Figure 5E).

### 2.6. Molecular Morphology

**Figure 6:**
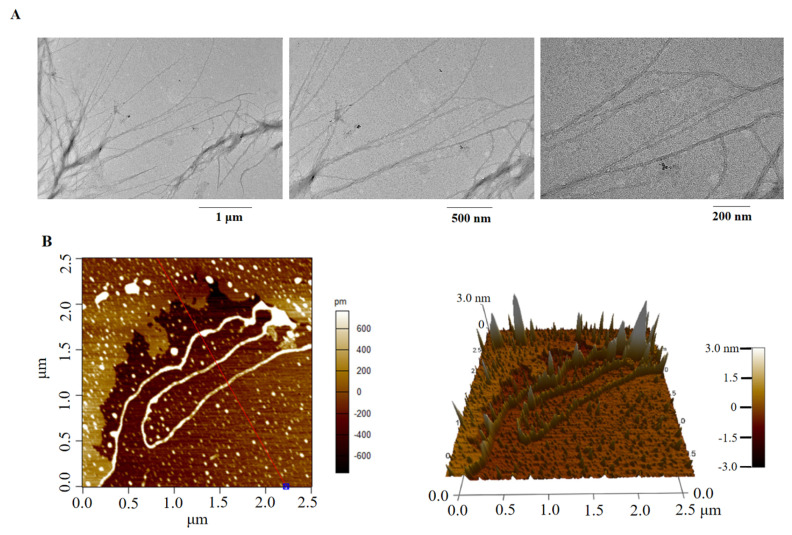
Morphological analysis of EPS2-1. (**A**) TEM images (12,000×, 20,000×, 40,000×); (**B**) AFM images of EPS2-1.

Molecular morphology is used to demonstrate the structure of macromolecules intuitively. A main linear chain with side chains was enriched in EPS2-1, which was in agreement with the results of GC-MS and NMR analysis. Thus, EPS2-1 was a branched polysaccharide [26]. The linear chains of EPS2-1 were entangled together with each other, which made EPS2-1 look like a bow tie (Figure 6A) [27]. It has been reported that the entangled chains were beneficial for improving the biological activities of glycans [28].

Atomic force microscopy (AFM) is an effective method to observe conformation and provide three-dimensional structure information of macromolecules [29]. AFM revealed that EPS2-1 exhibits chain conformation of regular line clusters (Figure 6B). The height of single-chain polysaccharides was usually between 0.1 and 1.0 nm, and the height of EPS2-1 ranged from −3.0 nm to 3.0 nm. AFM indicated that EPS2-1 has branches and may interweave with each other due to van der Waals and other forces between molecular chains [30].

### 2.7. In Vitro Antioxidant Activities of EPS2-1

Free radicals can trigger the progression of senescence-related diseases, such as Alzheimer’s disease, diabetes, and cancer. Free radicals can accept electrons or hydrogen atoms from antioxidants to get a stable status [31]. Therefore, antioxidants are widely used in the pharmaceutical and food industries. Hence, DDPH, ABTS, hydroxyl radicals, and FRAP assays were used to evaluate the antioxidant activity of EPS2-1 in this study [32].

**Figure 7:**
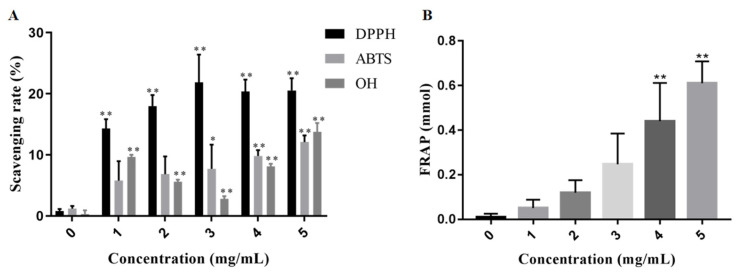
In vitro antioxidant activities of EPS2-1. (**A**) DDPH, ABTS, hydroxyl free radicals scavenging ability; (**B**) FRAP assay of EPS2-1. Data are presented as mean ± SD compared with the control group, * *p* < 0.05 and ** *p* < 0.01.

The scavenging effects of EPS2-1 on free radicals are illustrated in Figure 7A. The DPPH-scavenging rate of EPS2-1 in this study was as high as 20.55% at the concentration of 5 mg/mL. EPS2-1 scavenged ABTS in a dose-dependent manner, at the concentration of 5 mg/mL, the scavenging rate reached 12.09%. The scavenging rate of the hydroxyl radical by EPS2-1 at 5 mg/mL was 13.76%. Antioxidants can reduce Fe^3+^ to Fe^2+^, hence, FRAP assay was used to detect the reducing power of antioxidants. The reducing power of EPS2-1 was shown in Figure 7B and indicated by the FRAP value. With the increase of EPS2-1 concentration, the FRAP value was increased in a concentration-dependent manner. The FRAP value of EPS2-1 ranged from 0.051 mmol to 0.610 mmol, the FRAP value at 4 mg/mL and 5 mg/mL were significantly higher than the control group.

### 2.8. Effect of EPS2-1 in the Proliferation of Tumor and RAW264.7 Cells

**Figure 8:**
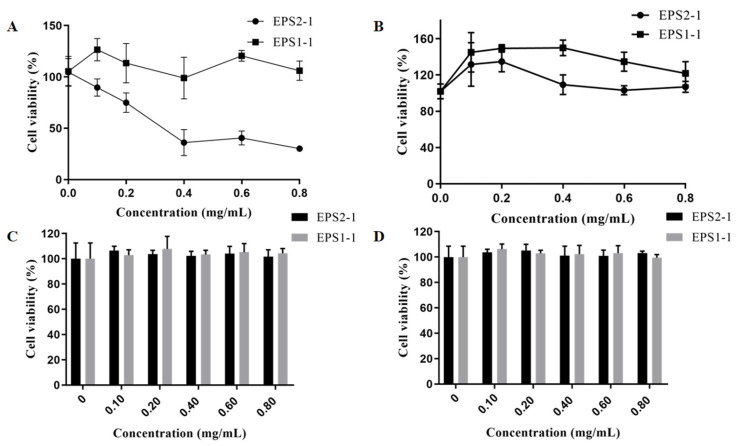
Effect of EPS2-1 on the proliferation of tumor and RAW264.7 cells. (**A**) MFC cells; (**B**) RAW264.7 cells; (**C**) SPCA1 cells; (**D**) H1299 cells were treated with EPS2-1 for 24 h, and the cell viability was detected by CCK-8 assay. Data were presented as mean ± SD.

To explore the potential immunomodulatory and antitumor activities of EPS2-1, tumor and RAW264.7 cells were treated with EPS2-1 for 24 h. Then, the CCK-8 assay was used to detect the effect of EPS2-1 on cell viability. As shown in Figure 8A, EPS2-1 significantly decreased the cell viability of MFC cells, where the viability of MFC cells was 89.68%, 74.99%, 36.09%, and 40.60% at concentrations ranging from 0.1 to 0.8 mg/mL. At the same time, EPS2-1 and EPS1-1 could not affect the cell viability of human lung cancer cells SPCA1 and H1299 (*p* > 0.05) (Figure 8C,D). Furthermore, EPS2-1 and EPS1-1 (0.1 to 0.8 mg/mL) could increase the cell viability of RAW264.7 cells (Figure 8B). Compared with the control group, EPS2-1 significantly increased the cell viability of RAW264.7 cells by up to 135.04% at 0.2 mg/mL.

## 3. Materials and Methods

### 3.1. Materials and Reagents

*R. Nigricans* (CGMCC No. 8436) was preserved in the State Key Laboratory of Microbial Technology, Shandong University. DEAE-52, Sephadex G-100, Sephadex G-75 column, Cell Counting Kit (CCK-8), and DMSO solution were purchased from Solarbio Life Sciences (Beijing, China). All standard monosaccharides were supplied from Sigma-Aldrich (St. Louis, MO, USA). 1,1-diphenyl-2-picrylhydrazyl (DPPH), 3-ethylbenzothiazoline-6-sulfonic acid (ABTS), FRAP assay kit, and hydroxyl radicals assay kit were purchased from Nanjing Jiancheng Bioengineering Institute (Nanjing, China). DMEM medium was obtained from Beyotime Biotechnology Co. (Shanghai, China).

### 3.2. Isolation and Purification of Exopolysaccharides from Rhizopus Nigricans

The crude exopolysaccharides were extracted from *R. nigricans* according to previously reported methods [33]. Briefly, *R. nigricans* was cultured in Potato Dextrose Broth (PDB) for 7 d at 28 °C, then the fermentation liquor of *R. nigricans* was precipitated with four volumes of ethanol for 48 h. The crude polysaccharide was obtained after deproteinization, decoloration, dialysis, and lyophilization. The lyophilized polysaccharide powder was dissolved in 0.02 M Tris–HCl (pH 7.4), which was purified by column chromatography of DEAE-52 Sepharose, followed by Tris–HCl buffer and a stepwise elution using NaCl (0 and 0.3 M) at a flow rate of 1 mL/min [34]. Two buffer-eluted fractions were collected, and then purified through Sephadex G-100 and Sephadex G-75 columns with ultra-pure water. The major peaks were collected and lyophilized, named EPS1-1 and EPS2-1, respectively [35]. Finally, total carbohydrate content was measured by the phenol–sulfuric acid method [36,37].

### 3.3. Molecular Weight Determination

The Mw of EPS2-1 was determined using HPGPC through the LC-20A system (Shimadzu, Kyoto, Japan) equipped with TSKgel G2500PWXL gel column (Tosoh Corp., Yamaguchi, Japan) and RID-10A detector. In addition, a series of dextrans standards (5, 11.6, 23.8, 48.6, 80.9 kDa) were used to construct the standard curve. Samples of 20 μL (5 mg/mL) were injected, then eluted with distilled water (0.6 mL/min) [38].

### 3.4. Monosaccharide Composition Analysis

High-performance ion exchange chromatography (HPIC) was used to analyze the monosaccharide composition of EPS2-1, the experimental methods referred to the previously reported methods [39]. Briefly, EPS2-1 was hydrolyzed using 10 mL TFA (3 M). After dissolving in methanol to remove residual TFA, the dried hydrolysates were dissolved in distilled water and injected into HPIC. The HPIC system was equipped with Dionex Carbopac PA20 column (ICS5000, 150 mm × 3.0 mm × 10 μm, Thermo Fisher Scientific, Co., Ltd., MA, USA), with an injection volume of 5 μL.

### 3.5. FT-IR and UV Spectroscopy

The FT-IR spectra of EPS2-1 (KBr pellets) was obtained using a Nicolet 10 spectrometer (Thermo Fisher Scientific, Co., Ltd., MA, USA) in the frequency range of 4000–500 cm^−1^. A 5 mg sample of EPS2-1 was dissolved in 5 mL of distilled water, and then the sample solution was filtered through a 0.22 μm filtration membrane. Then, the UV spectroscopy of EPS2-1 was analyzed using an Ultrospec 7000 spectrometer (GE, MA, USA) [40].

### 3.6. Methylation and GC-MS Analysis

The methylation and GC-MS analysis were performed according to the previously reported method [21]. Briefly, EPS2-1 was dissolved in 1 mL of DMSO, and 20 mg sodium hydroxide was added under nitrogen protection. Then 3.6 mL of methyl iodide was added dropwise, and the reaction was quenched with 2 mL distilled water. The methylated product was hydrolyzed with 1 mL of trifluoroacetate (2 M, 121 °C, 90 min). Next, the hydrolysis sample was reduced with 1 M NaBH_4_. Acetic acid was used to stop the reaction, then 0.25 mL of acetic anhydride was added. Finally, the acetylation sample was dissolved in 3 mL of chloroform, and the dichloromethane phase was harvested. The obtained product was detected using the GC-MS-QP 2010 system (Shimadzu, Kyoto, Japan), which was equipped with a RXI-5 SIL MS column.

### 3.7. NMR Analysis

A sample of 50 mg of EPS2-1 was dissolved in 0.5 mL of D_2_O for repeated freeze-drying, and the treated samples were dissolved in 0.5 mL of D_2_O [41]. The 1D NMR (^1^H NMR and ^13^C NMR) and 2D NMR of EPS2-1 were analyzed using the Bruker Avance 600 MHz NMR instrument. The 2D NMR included HSQC spectroscopy, HMBC spectroscopy, COSY spectroscopy, and NOESY spectroscopy.

### 3.8. Morphological Analysis

For transmission electron microscopy (TEM), 1 mL of EPS2-1 (1 μg/mL) was filtered through the 0.22 μm filtration membrane. Next, 10 μL of the solution was transferred to a 400-mesh copper grid (carbon-coated) and dried naturally [42]. Then, the sample was observed in JEM 2100F (JOEL, Tokyo, Japan). For the atomic force microscope (AFM) analysis, 10 μL of EPS2-1 (1 μg/mL) was added to the surface of mica flakes, then the sample was analyzed through an AFM (Bruker, Karlsruhe, Germany).

### 3.9. Antioxidant Activity In Vitro

The DPPH radical assay, ABTS radical assay, hydroxyl radical assay, and the ferric reducing antioxidant power (FRAP) assay were measured according to the previously described methods [43,44]. For the DPPH radical scavenging assay, 0.25 mL of the sample and 1.25 mL of DPPH–ethanol solution (0.3 mM) were incubated in the dark at 28 °C for 30 min. Then, the absorbance was measured at 515 nm [45]. For FRAP assay, the absorbance of the reaction solution at 593 nm represented the FRAP of samples [46].

### 3.10. Cell Culture

MFC, RAW264.7, SPCA1, and H1299 cells were cultured in DMEM medium (high glucose) containing 10% fetal bovine serum, 100 U/mL penicillin, and 110 μg/mL streptomycin at 37 °C in the environment containing 5% CO_2_ [47].

### 3.11. Cell Viability Assay

Cells were seeded in 96-well plates (6 × 10^3^ cells/well) for 24 h. After being treated with polysaccharides for 24 h, the CCK-8 reagent was added. The absorbance was detected at 450 nm using Microplate Reader (BioTek, Winooski, VT, USA).

### 3.12. Statistical Analysis

The difference between groups was evaluated by *t*-test and one-way ANOVA through GraphPad Prism 8.0. *p* values < 0.05 were considered statistically significant. All results were expressed as mean ± SD.

## 4. Conclusions

In this study, a novel heteropolysaccharide (EPS2-1) was isolated and purified from the fermentation broth of *R. nigricans*. The Mw of EPS2-1 was 32.803 kDa, and EPS2-1 was composed of Man, Gal, Glc, Ara, and Fuc in the ratio of 0.519:0.204:0.065:0.031 and 0.029. The results of monosaccharide composition, methylation analysis, and NMR analysis indicated that EPS2-1 is a heteroglycan with the main chain →2)-α-D-Manp-(1→2,6)-α-D-Manp-(1→3,6)-β-D-Galp-(1→3,6)-β-D-Galp-(1→, and the branched chain α-D-Manp-(1→6)-α-D-Manp-(1→, →4)-β-D-Glcp-(1→ and →3)-β-D-Galp-(1→. Antioxidant activity assays showed that EPS2-1 keeps a certain scavenging activity on DPPH, ABTS, and hydroxyl radicals, and shows iron ion reducing abilities, which suggested that EPS2-1 has antioxidant activity. In addition, we found that EPS2-1 inhibits the proliferation of MFC cells and increases the cell viability of RAW 264.7 cells, and the inhibitory effect on MFC cells of EPS2-1 is better than EPS1-1. Furthermore, EPS2-1 could not affect the cell viability of human lung cancer cells SPCA1 and H1299. To sum up, EPS2-1 is a novel polysaccharide with antioxidant activity, potential immunomodulatory, and anticancer activities. This study unraveled the promising medicinal use as natural antioxidant and anticancer agents, further research should be carried out to understand the antioxidant and antitumor activity in vivo.

## Figures and Tables

**Table 1:** Monosaccharide composition analysis of EPS2-1.

Retention Time (min)	Monosaccharide Composition	Relative Molar Ratio
5.844	fucose	0.029
12.592	arabinose	0.031
15.775	galactose	0.204
17.875	glucose	0.065
22.084	mannose	0.519
11.567	rhamnose	0.000
20.867	xylose	0.000
25.159	fructose	0.000
27.95	ribose	0.000
44.25	galacturonic acid	0.000
44.942	guluronic acid	0.000
47.017	glucuronic acid	0.000
49.742	mannuronic acid	0.000

**Table 2:** Results of methylation analysis.

RT	Methylated Sugar	Mass Fragments (*m*/*z*)	Molar Ratios	Type of Linkage
16.253	2,3,4,6-Me_4_-Man*p*	43,71,87,101,117,129,145,161,205	3.42	Man*p*-(1→
20.580	3,4,6-Me_3_-Man*p*	43,71,87,99,101,129,145,161,189	64.37	→2)-Man*p*-(1→
20.843	2,3,6-Me_3_-Glc*p*	43,71,87,99,101,113,117,129,131,161,173,233	4.35	→4)-Glc*p*-(1→
21.471	2,4,6-Me_3_-Man*p*	43,71,87,99,101,117,129,161,201,233	2.02	→3)-Man*p*-(1→
21.983	2,4,6-Me_3_-Gal*p*	43,71,87,101,117,129,143,161,173,233	1.68	→3)-Gal*p*-(1→
22.181	2,3,4-Me_3_-Glc*p*	43,71,87,99,101,117,129,161,173,189,233	1.69	→6)-Glc*p*-(1→
22.372	2,3,4-Me_3_-Man*p*	43,71,87,99,101,117,129,161,173,189	12.09	→6)-Man*p*-(1→
24.228	3,4–Me_2_-Man*p*	43,71,87,99,129,159,173,189,233	1.41	→2,6)-Man*p*-(1→
29.312	2,4-Me_2_-Gal*p*	43,87,101,117,129,139,159,173,189,233	8.97	→3,6)-Gal*p*-(1→

**Table 3:** ^13^C-NMR and ^1^H-NMR spectral assignments of EPS2-1.

Glycosyl Residues	C1/H1	C2/H2	C3/H3	C4/H4	C5/H5	C6/H6a	H6b
(A):→3)-β-D-Gal*p*-(1→	104.48/4.37	71.97/3.47	81.58/3.60	70.25/3.97	76.40/3.64	62.26/3.69	
(B):→2)-α-D-Man*p*-(1→	101.95/5.23	80.21/4.01	71.32/3.88	68.19/3.6	74.5/3.7	62.3/3.69	3.81
(D):→3,6)-β-D-Gal*p*-(1→	104.49/4.47	71.31/3.57	81.5/3.68	69.82/4.05	74.81/3.87	70.76/3.96	3.86
(E):→6)-α-D-Man*p*-(1→	100.83/4.85	71.42/3.94	71.95/3.8	68.03/3.65	74.53/3.78	67.2/3.78	3.71
(F):→4)-β-D-Glc*p*-(1→	103.84/4.46	74.17/3.3	71.58/3.63	80.16/3.64	76.69/3.47	61.67/3.95	3.79
(G):α-D-Man*p*-(1→	103.6/4.99	71.44/4.01	71.95/3.78	68.66/3.58	74.67/3.71	62.4/3.68	3.82
(I):→6)-α-D-Glc*p*-(1→	99.1/4.91	72.73/3.53	74.63/3.67	70.87/3.47	71.47/3.86	66.8/3.7	3.93
(J):→2,6)-α-D-Man*p*-(1→	99.62/5.05	80.12/3.98	71.58/3.86	68.06/3.6	74.52/3.7	67.2/3.77	3.72
(K):→3)-α-D-Man*p*-(1→	101.1/5.15	71.55/3.65	79.7/3.88	68.4/3.6	74.75/3.78	62.16/3.69	3.82

## Data Availability

Not applicable.
